# Supplementary material for: Phylogenomics and barcoding of Panax: toward the identification of ginseng species
Source: BMC Evol Biol. 2018 Apr 3;18:44. doi: 10.1186/s12862-018-1160-y (PMC5883351; doi:10.1186/s12862-018-1160-y)
Supplement: Supplementary file 1 — Table S1. Voucher specimens. (DOCX 14 kb) [file 12862_2018_1160_MOESM1_ESM.docx]

**SAMPLING INFORMATION**

| Sample | Voucher number | Submited to | Collected location | Collection date | Collector  identification | Biosample |
| --- | --- | --- | --- | --- | --- | --- |
| *Panax vietnamensis* Ha et Grushv. | VMN-B 2148 | Vietnam National Museum of Nature (VNMN) | Location: Tra Linh Medicinal Plant Center, Nam Tra My, Quang Nam province  Height: 1835 m  Coordinate: 15^o^01.906’N  107^o^58.746’E | 22/10/2015 | Nguyễn Văn Viễn  (Nguyen Van Vien) | [SRS2302306](https://trace.ncbi.nlm.nih.gov/Traces/sra_sub/sub.cgi?acc=SRS2302306&focus=SRS2302306&from=list&action=show:SAMPLE) |
| *Panax bipinnatifidus* | APB57.1 | Vietnam National University of Forestry (VNUF) | Location: Khoang Village, Sa Pa, Lao Cai province  Height: 1476 m  Coordinate: 22°24'08"N 103°48'25"E | 25/8/2015 | Nguyễn Tiến Dũng  (Nguyen Tien Dung) | [SRS2302308](https://trace.ncbi.nlm.nih.gov/Traces/sra_sub/sub.cgi?acc=SRS2302308&focus=SRS2302308&from=list&action=show:SAMPLE) |
| *Panax stipuleanatus* | APS58.2 | Vietnam National University of Forestry (VNUF) | Location: Khoang village, Sa Pa, Lao Cai province  Height: 1518 m  Coordinate: 22°23'48"N 103°46'51"E | 26/8/2015 | Nguyễn Tiến Dũng  (Nguyen Tien Dung) | [SRS2302559](https://trace.ncbi.nlm.nih.gov/Traces/sra_sub/sub.cgi?acc=SRS2302559&focus=SRS2302559&from=list&action=show:SAMPLE) |
| *Panax sp.* (puxailaileng) | VMN-B 2172 | Vietnam National Museum of Nature (VNMN) | Location: Muong Long, Ky Son, Nghe An province  Height: 1327 m  Coordinate: 19°31'50"N 104°20'20"E | 7/12/2015 | Nguyễn Tiến Dũng  (Nguyen Tien Dung) | [SRS2302307](https://trace.ncbi.nlm.nih.gov/Traces/sra_sub/sub.cgi?acc=SRS2302306&focus=SRS2302306&from=list&action=show:SAMPLE) |
