# Supplementary material for: Phylogenomics and barcoding of Panax: toward the identification of ginseng species
Source: BMC Evol Biol. 2018 Apr 3;18:44. doi: 10.1186/s12862-018-1160-y (PMC5883351; doi:10.1186/s12862-018-1160-y)
Supplement: Supplementary file 2 — Table S2. Araliaceae species used for this study and their accession numbers. (PDF 385 kb) [file 12862_2018_1160_MOESM2_ESM.pdf]

NCBI Reference Sequence	Genus	Species	Cultivar	Reference article
KT153023	*Aralia*	*elata*		[1]
NC_022810	*Aralia*	*undulata*		[2]
KC456163	*Aralia*	*undulata*		[3, 4]
NC_022811	*Brassaiopsis*	*hainla*		[4]
KC456164	*Brassaiopsis*	*hainla*		[3, 4]
KP271241	*Dendropanax*	*dentiger*		[3, 5]
NC_026546	*Dendropanax*	*dentiger*		[6]
KR136270	*Dendropanax*	*morbifer*		[1]
NC_027607	*Dendropanax*	*morbifer*		[7]
KT153020	*Eleutherococcus*	*gracilistylus*		NA
JN637765	*Eleutherococcus*	*senticosus*		[3, 4]
NC_016430	*Eleutherococcus*	*senticosus*		[1, 8]
NC_027685	*Fatsia*	*japonica*		[9]
KR021045	*Fatsia*	*japonica*		[9]
HM596070	*Hydrocotyle*	*verticillata*		[3, 5]
KC456167	*Kalopanax*	*septemlobus*		[3-5]
NC_022814	*Kalopanax*	*septemlobus*		[4]
NC_022812	*Metapanax*	*delavayi*		[4]
KC456165	*Metapanax*	*delavayi*		[3, 4]
MF377620	*Panax*	*bipinnatifidus*		This article.
KM067386	*Panax*	*ginseng*	Cheongsun	[10]
KM088019	*Panax*	*ginseng*	Chunpoong	[1, 10]
KC686331	*Panax*	*ginseng*	damaya	[10, 11]
KC686332	*Panax*	*ginseng*	Ermaya	[3, 10, 11]
KC686333	*Panax*	*ginseng*	gaolishen	[10, 11]
KM067387	*Panax*	*ginseng*	Gopoong	[10]
KM067388	*Panax*	*ginseng*	Gumpoong	[10]
KM067394	*Panax*	*ginseng*	Hwangsook	[10]
KM067389	*Panax*	*ginseng*	Jakyung	[10]
KM067393	*Panax*	*ginseng*	Sunhyang	[10]
KM067390	*Panax*	*ginseng*	Sunone	[10]
KM067391	*Panax*	*ginseng*	Sunpoong	[10]
KM067392	*Panax*	*ginseng*	Sunun	[10]
KM088020	*Panax*	*ginseng*	Yunpoong	[10]
AY582139	*Panax*	*ginseng*		[4, 5]
NC_006290	*Panax*	*ginseng*		[10, 11]
KF431956 [3]	*Panax*	*ginseng*		[3, 11]
KP036469	*Panax*	*japonicus*		[1]
NC_028703	*Panax*	*japonicus*		[1]
KJ566590	*Panax*	*notoginseng*		[3, 12]

NC_026447	*Panax*	*notoginseng*		[12]
KP036468	*Panax*	*notoginseng*		[1]
KR021381	*Panax*	*notoginseng*		NA
KT001509	*Panax*	*notoginseng*		NA
KT028714	*Panax*	*quinquefolius*		NA
KM088018	*Panax*	*quinquefolius*		[1, 10]
NC_027456	*Panax*	*quinquefolius*		[10]
NC_030598	*Panax*	*stipuleanatus*		NA
KX247147	*Panax*	*stipuleanatus*		NA
MF377622	*Panax*	*stipuleanatus*		This article.
MF377621	*Panax*	*vietnamensis*		This article.
MF377623	*Panax*	*vietnamensis*		This article.
KU059178	*Panax*	*vietnamensis*		[13]
KP036470	*Panax*	*vietnamensis*		[1]
KP036471	*Panax*	*vietnamensis*		[1]
NC_028704	*Panax*	*vietnamensis*		[1]
KC456166	*Schefflera*	*delavayi*		[3, 4]
NC_022813	*Schefflera*	*delavayi*		[4]
NC_029764	*Schefflera*	*heptaphylla*		[14]
KT748629	*Schefflera*	*heptaphylla*		[14]

1. Kim K, Nguyen VB, Dong J, Wang Y, Park JY, Lee S-C, Yang T-J: **Evolution of the Araliaceae family inferred from complete chloroplast genomes and 45S nrDNAs of 10 Panax-related species**. *Sci Rep* 2017, **7**:4917.
2. Bock DG, Kane NC, Ebert DP, Rieseberg LH: **Genome skimming reveals the origin of the Jerusalem Artichoke tuber crop species: neither from Jerusalem nor an artichoke**. *New Phytologist* 2014, **201**(3):1021-1030.
3. Shi F-X, Li M-R, Li Y-L, Jiang P, Zhang C, Pan Y-Z, Liu B, Xiao H-X, Li L-F: **The impacts of polyploidy, geographic and ecological isolations on the diversification of *Panax* (Araliaceae)**. *BMC Plant Biology* 2015, **15**(1):297.
4. Li R, Ma P-F, Wen J, Yi T-S: **Complete Sequencing of Five Araliaceae Chloroplast Genomes and the Phylogenetic Implications**. *PLOS ONE* 2013, **8**(10):e78568.
5. Yao X, Liu Y-Y, Tan Y-H, Song Y, Corlett RT: **The complete chloroplast genome sequence of Helwingia himalaica (Helwingiaceae, Aquifoliales) and a chloroplast phylogenomic analysis of the Campanulidae**. *PeerJ* 2016, **4**:e2734.
6. Wang L, Du X-J, Li X-F: **The complete chloroplast genome sequence of the evergreen plant Dendropanax dentiger (Araliaceae)**. *Mitochondrial DNA Part A* 2016, **27**(6):4193-4194.
7. Kim K, Lee S-C, Yang T-J: **The complete chloroplast genome sequence of Dendropanax morbifera (Léveillé)**. *Mitochondrial DNA Part A* 2016, **27**(4):2923-2924.
8. Yang J-B, Yang S-X, Li H-T, Yang J, Li D-Z: **Comparative chloroplast genomes of Camellia species**. *PLoS One* 2013, **8**(8):e73053.

9. Chen Q, Feng X, Li M, Yang B, Gao C, Zhang L, Tian J: **The complete chloroplast genome sequence of Fatsia japonica (Apiales: Araliaceae) and the phylogenetic analysis**. *Mitochondrial DNA Part A* 2016, **27**(4):3050-3051.
10. Kim K, Lee S-C, Lee J, Lee HO, Joh HJ, Kim N-H, Park H-S, Yang T-J: **Comprehensive Survey of Genetic Diversity in Chloroplast Genomes and 45S nrDNAs within Panax ginseng Species**. *PLOS ONE* 2015, **10**(6):e0117159.
11. Zhao Y, Yin J, Guo H, Zhang Y, Xiao W, Sun C, Wu J, Qu X, Yu J, Wang X *et al*: **The complete chloroplast genome provides insight into the evolution and polymorphism of Panax ginseng**. *Frontiers in Plant Science* 2015, **5**.
12. Dong W, Liu H, Xu C, Zuo Y, Chen Z, Zhou S: **A chloroplast genomic strategy for designing taxon specific DNA mini-barcodes: a case study on ginsengs**. *BMC genetics* 2014, **15**(1):138.
13. Nguyen B, Kim K, Kim Y-C, Lee S-C, Shin JE, Lee J, Kim N-H, Jang W, Choi H-I, Yang T-J: **The complete chloroplast genome sequence of *Panax vietnamensis* Ha et Grushv (Araliaceae)**. *Mitochondrial DNA Part A* 2017, **28**(1):85-86.
14. Zong X, Song J, Lv J, Wang S: **The complete chloroplast genome sequence of Schefflera octophylla**. *Mitochondrial DNA Part A* 2016, **27**(6):4685-4686.
