# Supplementary material for: Phylogenomics and barcoding of Panax: toward the identification of ginseng species
Source: BMC Evol Biol. 2018 Apr 3;18:44. doi: 10.1186/s12862-018-1160-y (PMC5883351; doi:10.1186/s12862-018-1160-y)
Supplement: Supplementary file 3 — Figure S1. Fragment analyzer DNA report of P. bipinnatifidus, P. sp. (puxailaileng), P. stipuleanatus, P. vietnamensis samples, for the genomic DNA (gDNA), for the non-methylated and methylated fractions. (PDF 410 kb) [file 12862_2018_1160_MOESM3_ESM.pdf]

	gDNA		Non-methylated		Methylated
1 P. sp (puxailaileng) 10.6465 ng/uL	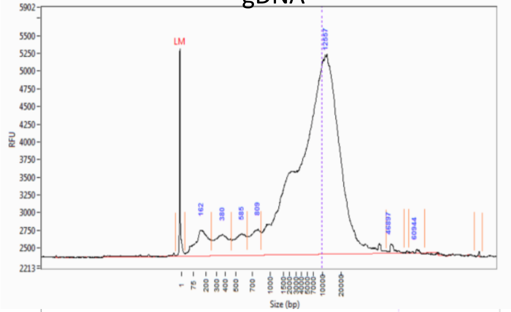	2.0033 ng/uL	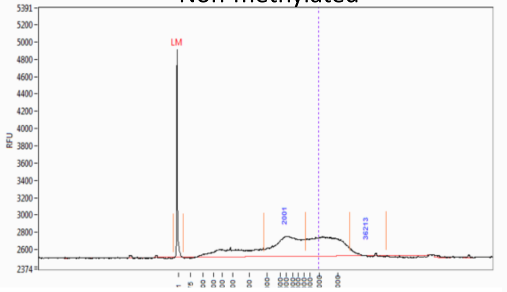	3.5500 ng/uL	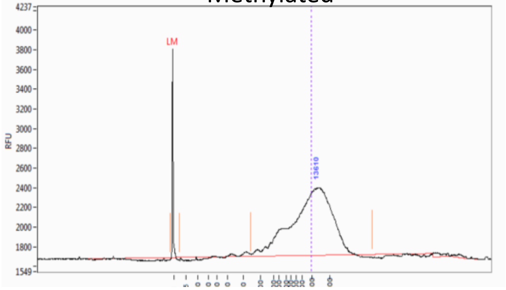
2 P. bipinnatifidus 4.7763 ng/uL	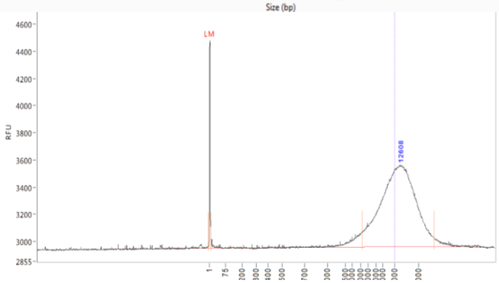	0.5657 ng/uL	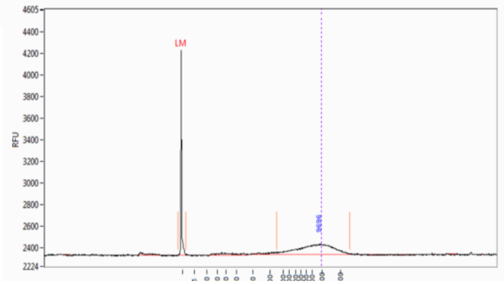	3.6451 ng/uL	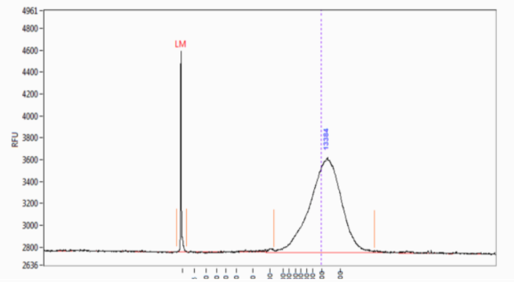
3 P. stipuleanatus 7.7039 ng/uL	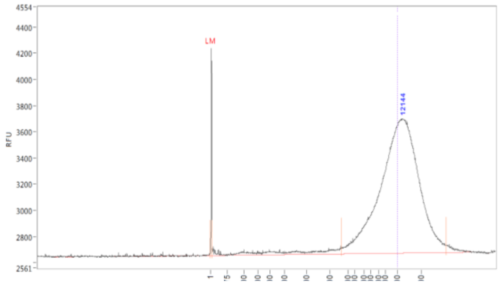	0.5132 ng/uL	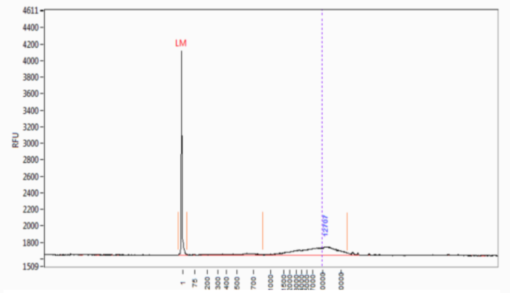	2.7907 ng/uL	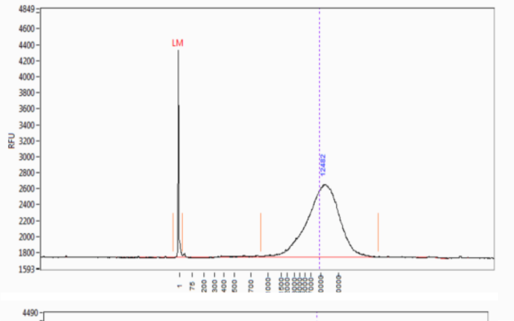
4 P. vietnamensis 4.93 ng/uL	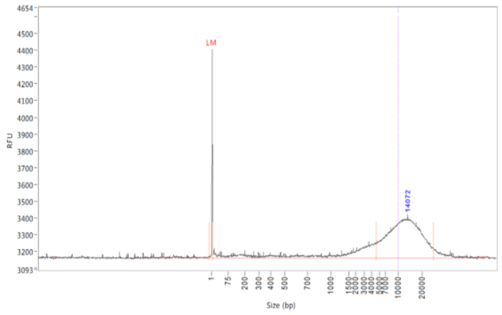	0.9733 ng/uL	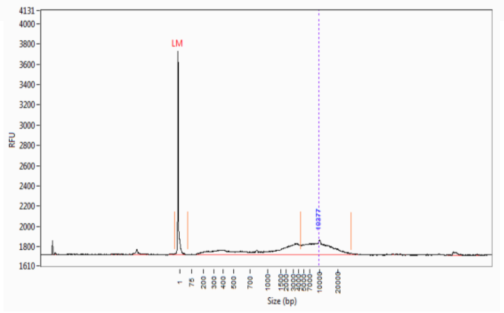	4.4691 ng/uL	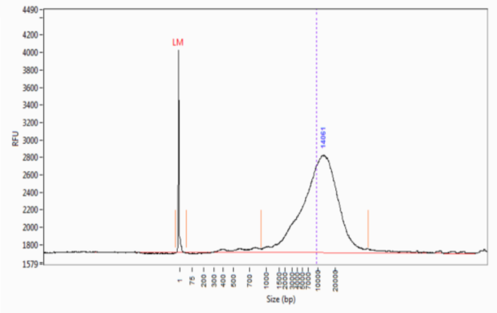
