# Supplementary material for: Phylogenomics and barcoding of Panax: toward the identification of ginseng species
Source: BMC Evol Biol. 2018 Apr 3;18:44. doi: 10.1186/s12862-018-1160-y (PMC5883351; doi:10.1186/s12862-018-1160-y)
Supplement: Supplementary file 4 — Table S3. Partition finder scheme. (DOCX 29 kb) [file 12862_2018_1160_MOESM4_ESM.docx]

**Table S3.** Partition finder scheme.

| Subset | Best Model | # sites | Partition names |
| --- | --- | --- | --- |
| 1 | GTR+I+G+X | 662 | *intron_1, intron_77* |
| 2 | GTR+G+X | 1316 | *psbA, psbE* |
| 3 | GTR+G+X | 1757 | *intron_16, intron_69, intron_3, intron_51* |
| 4 | GTR+G+X | 2219 | *intron_75, intron_31, intron_38, matK* |
| 5 | GTR+I+G+X | 1856 | *intron_4* |
| 6 | GTR+I+G+X | 5510 | *rps16, rps19, atpF, intron_29, ndhA, intron_73* |
| 7 | GTR+G+X | 2414 | *intron_72, intron_115, intron_5* |
| 8 | GTR+X | 2663 | *trnI-GAU, trnI-GAU, trnV-GAC, trnS-UGA, rrn5, trnC-GCA, intron_130, intron_128, trnC-GCA* |
| 9 | GTR+X | 1239 | *intron_30, intron_54, intron_63, intron_6* |
| 10 | GTR+G+X | 2214 | *psbZ, psbD, rps12, intron_97, psbK, petG, petL, petN, psbI* |
| 11 | GTR+G+X | 1860 | *intron_80, intron_110, intron_7, intron_48, intron_123, rps19, intron_120* |
| 12 | GTR+G+X | 2729 | *intron_26, intron_111, rps19, intron_8, intron_41* |
| 13 | GTR+I+G+X | 2419 | *atpB, psaC, intron_101, trnS-GCU, intron_131* |
| 14 | GTR+G+X | 1725 | *intron_84, intron_74, intron_9, intron_119* |
| 15 | GTR+I+G+X | 3465 | *trnG-UCC, intron_19, intron_27, intron_85, intron_24* |
| 16 | GTR+X | 380 | *intron_10, psbM* |
| 17 | GTR | 988 | *trnS-GGA, trnN-GUU, rrn4_5, trnL-CAA, trnC-GCA, trnT-GGU, trnI-CAU, trnI-CAU, trnR-UCU, psbN, psbF* |
| 18 | GTR+I+G+X | 1649 | *rpl16, intron_11, intron_90* |
| 19 | GTR+G+X | 4693 | *intron_44, atpA, rps4, petA, rps2* |
| 20 | GTR+G+X | 2067 | *intron_13, intron_83, intron_20, intron_57, intron_21* |
| 21 | GTR+G+X | 5095 | *ndhC, atpH, psaA, psaB* |
| 22 | GTR+G+X | 1239 | *intron_14* |
| 23 | GTR+I+G+X | 2752 | *rpl33, atpI, ndhI, ndhJ, psbJ, rpl32, ndhG* |
| 24 | GTR+G+X | 2245 | *intron_43, intron_22, intron_15, intron_70* |
| 25 | GTR+I+G+X | 6513 | *intron_76, trnT-UGU, rpoC2, trnV-UAC, ndhH, infA* |
| 26 | GTR+G+X | 1256 | *psbT, atpE, rpl22, intron_17* |
| 27 | GTR+I+G+X | 2934 | *rpoC1* |
| 28 | GTR+G+X | 3218 | *rpoB* |
| 29 | GTR+X | 308 | *intron_64, psbM, rps19* |
| 30 | GTR+G+X | 3113 | *intron_23, intron_53, intron_61, intron_56* |
| 31 | GTR+I+G+X | 4948 | *rrn23, rrn16, rrn4_5_2, trnL-CAA, trnD-GUC, trnS-GGA, trnW-CCA, trnE-UUC, trnW-CCA, trnW-CCA* |
| 32 | GTR+I+G+X | 1550 | *rrn5, trnY-GUA, intron_102, trnG-UCC, trnV-GAC, trnA-UGC, trnL-UAG, intron_104, trnM-CAU* |
| 33 | GTR+I+G+X | 1519 | *petB, intron_25* |
| 34 | GTR+I+G+X | 3125 | *intron_124, psbC, psbB* |
| 35 | GTR+G+X | 2110 | *intron_52, intron_122, intron_32, intron_37, intron_112* |
| 36 | GTR+I+G+X | 1801 | *intron_34, ndhD, intron_33* |
| 37 | GTR+G+X | 2147 | *intron_99, rps14* |
| 38 | GTR+G+X | 2555 | *intron_78, intron_66, intron_50, intron_36* |
| 39 | GTR+I+G+X | 3993 | *psaI, rps3, rpl20, ycf3, cemA, rpl36* |
| 40 | GTR+X | 500 | *intron_39, intron_141* |
| 41 | GTR+I+G+X | 2249 | *petD, trnL-UAA, ndhE, rps19* |
| 42 | GTR+X | 996 | *intron_42, intron_65, intron_55, intron_47* |
| 43 | GTR+G+X | 1840 | *intron_46, intron_62, intron_114, intron_117* |
| 44 | GTR+X | 1975 | *rps18, trnT-GGU, psbL, psbT, intron_135, intron_96* |
| 45 | GTR+I+G+X | 1466 | *rbcL* |
| 46 | GTR+G+X | 2104 | *accD, ycf4* |
| 47 | GTR+G+X | 158 | *petL* |
| 48 | GTR+X | 919 | *rps11, intron_58, rpl14* |
| 49 | GTR+G+X | 344 | *intron_68* |
| 50 | GTR+I+G+X | 3130 | *rpoA, ccsA, rps15, intron_71* |
| 51 | GTR+I+G+X | 2106 | *clpP* |
| 52 | GTR+G+X | 910 | *rps8, rps19, psbH* |
| 53 | GTR+I+G+X | 288 | *intron_87, intron_79* |
| 54 | GTR+G+X | 113 | *intron_81* |
| 55 | GTR+X | 351 | *intron_82, intron_118* |
| 56 | GTR+G+X | 87 | *rps19* |
| 57 | GTR+I+G+X | 207 | *intron_89* |
| 58 | GTR+X | 3370 | *intron_105, intron_132, ycf15, rpl2, rpl23, intron_138, intron_129, rps7* |
| 59 | GTR+I+G+X | 2031 | *ycf1, intron_139, intron_92* |
| 60 | GTR+X | 2601 | *ndhB, rpl23, intron_93* |
| 61 | GTR+I+G+X | 13807 | *ycf2, intron_106, intron_108, ycf2, intron_126* |
| 62 | GTR+G+X | 3152 | *intron_133, intron_103, ycf15* |
| 63 | GTR+I+G+X | 319 | *intron_95* |
| 64 | GTR+X | 3009 | *ndhB, rps12* |
| 65 | GTR+X | 2725 | *intron_100, intron_127, intron_134, rpl2, rps7* |
| 66 | GTR+I+G+X | 4390 | *rrn23, rrn16, trnS-GGA* |
| 67 | GTR+I+G+X | 1254 | *intron_107, intron_125* |
| 68 | GTR+I+G+X | 2371 | *ndhF* |
| 69 | GTR+G+X | 970 | *intron_113* |
| 70 | GTR+I+G+X | 119 | *intron_116* |
| 71 | GTR+I+G+X | 6186 | *ycf1* |
| 72 | GTR+I+G+X | 887 | *trnA-UGC* |
| 73 | GTR+I+G+X | 319 | *intron_136* |
