# Supplementary material for: Phylogenomics and barcoding of Panax: toward the identification of ginseng species
Source: BMC Evol Biol. 2018 Apr 3;18:44. doi: 10.1186/s12862-018-1160-y (PMC5883351; doi:10.1186/s12862-018-1160-y)
Supplement: Supplementary file 5 — Table S4. selected markers and their primer sequences. (DOCX 19 kb) [file 12862_2018_1160_MOESM5_ESM.docx]

**Table S4**. selected markers and their primer sequences.

| **Name** | **Length primer** | **Direction** | **Primer sequence** | **Tm** | **Product size** |
| --- | --- | --- | --- | --- | --- |
| *trnC-rps16* | 25 | forward | GAAGATTTAGGTCCTTAGTCGTTCG | 59.3 | 738 |
|  | 24 | reverse | GATTCAGCATTCCCAGAGAATTGG | 60 | 738 |
| *trnS-trnG* | 20 | forward | GCCGCTTTAGTCCACTCAGC | 61.4 | 737 |
|  | 22 | reverse | GTGTTGACATTTTTCGTGGGGG | 60.5 | 737 |
| *petB* | 20 | forward | AATATTCAGACCTCGCGGCC | 60.3 | 593 |
|  | 20 | reverse | GGCTCAAGCAAAACACCCAA | 59.5 | 593 |
| *trnE-trnM* | 20 | forward | GAGTGGTTGGTCCGTCAGAA | 59.6 | 657 |
|  | 20 | reverse | CATGGCGTTACTCTACCGCT | 59.9 | 657 |
