# Supplementary figures and images for: Phylogenomics and barcoding of Panax: toward the identification of ginseng species
Source: BMC Evol Biol. 2018 Apr 3;18:44. doi: 10.1186/s12862-018-1160-y (PMC5883351; doi:10.1186/s12862-018-1160-y)

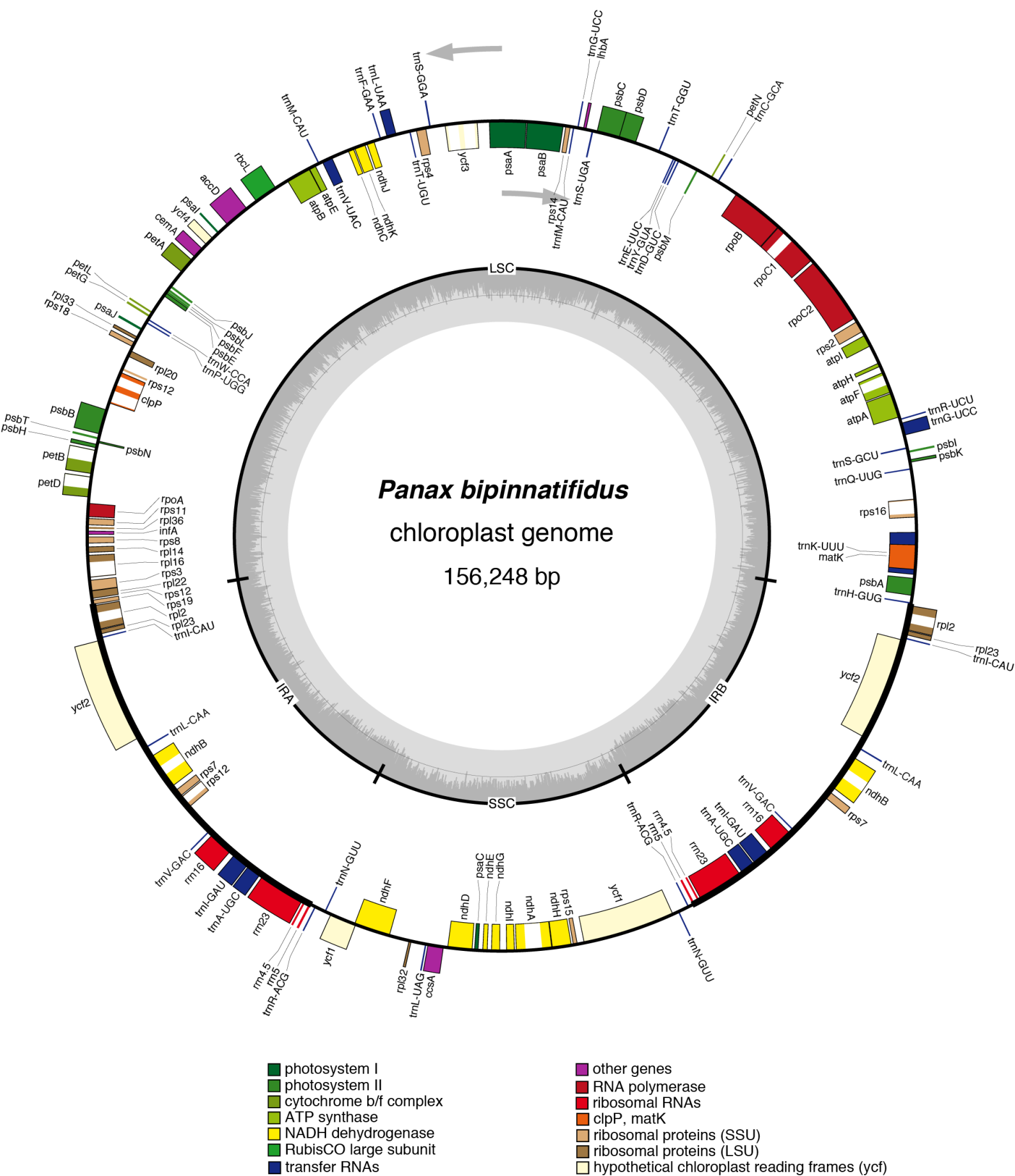

Supplement: Supplementary file 6 — Figure S2. Annotated plastid genome for P. binnatifidus (PDF 442 kb) [file 12862_2018_1160_MOESM6_ESM.pdf]

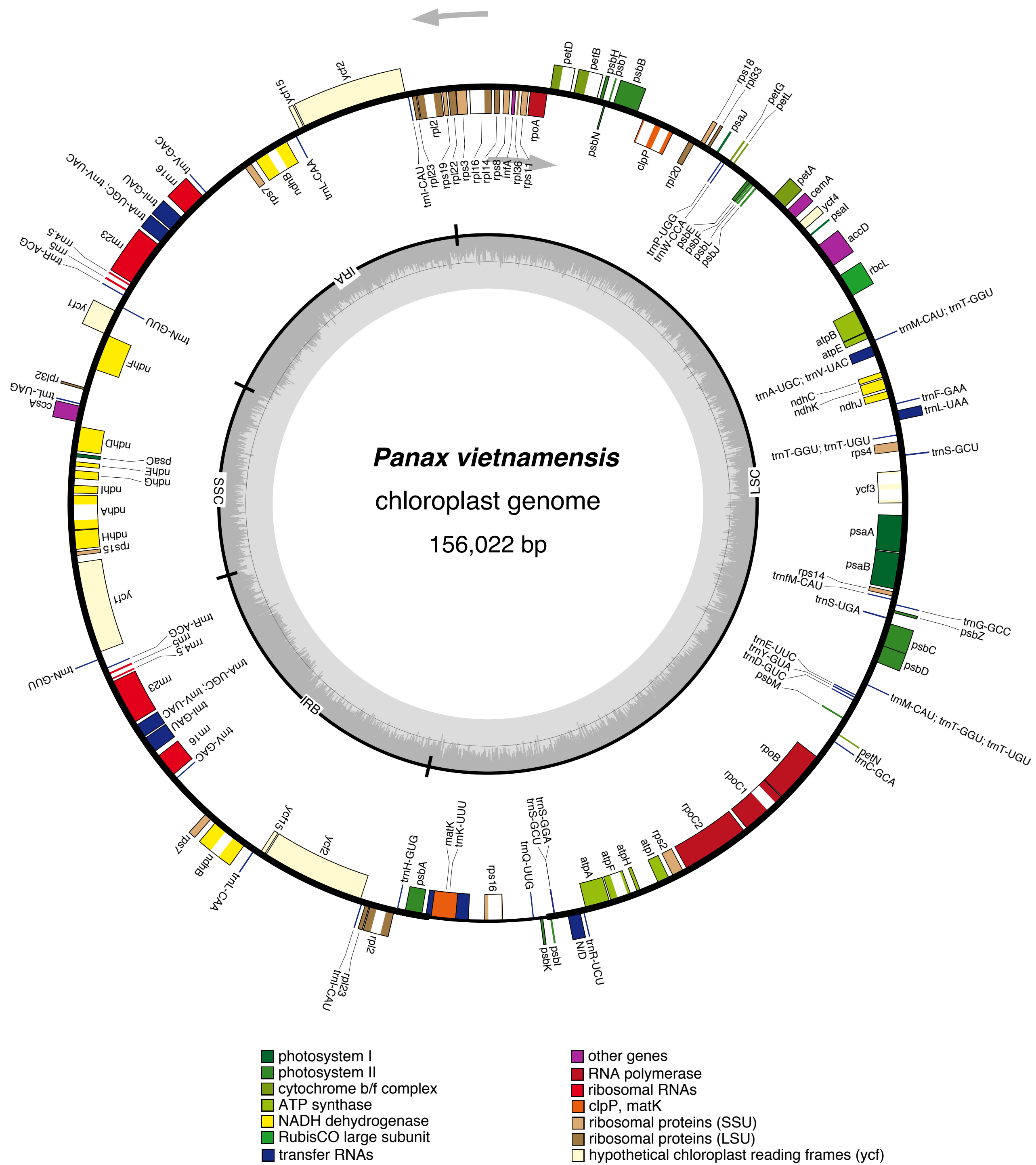

Supplement: Supplementary file 7 — Figure S3. Annotated plastid genome for P. sp. (puxailaileng). (PDF 359 kb) [file 12862_2018_1160_MOESM7_ESM.pdf]

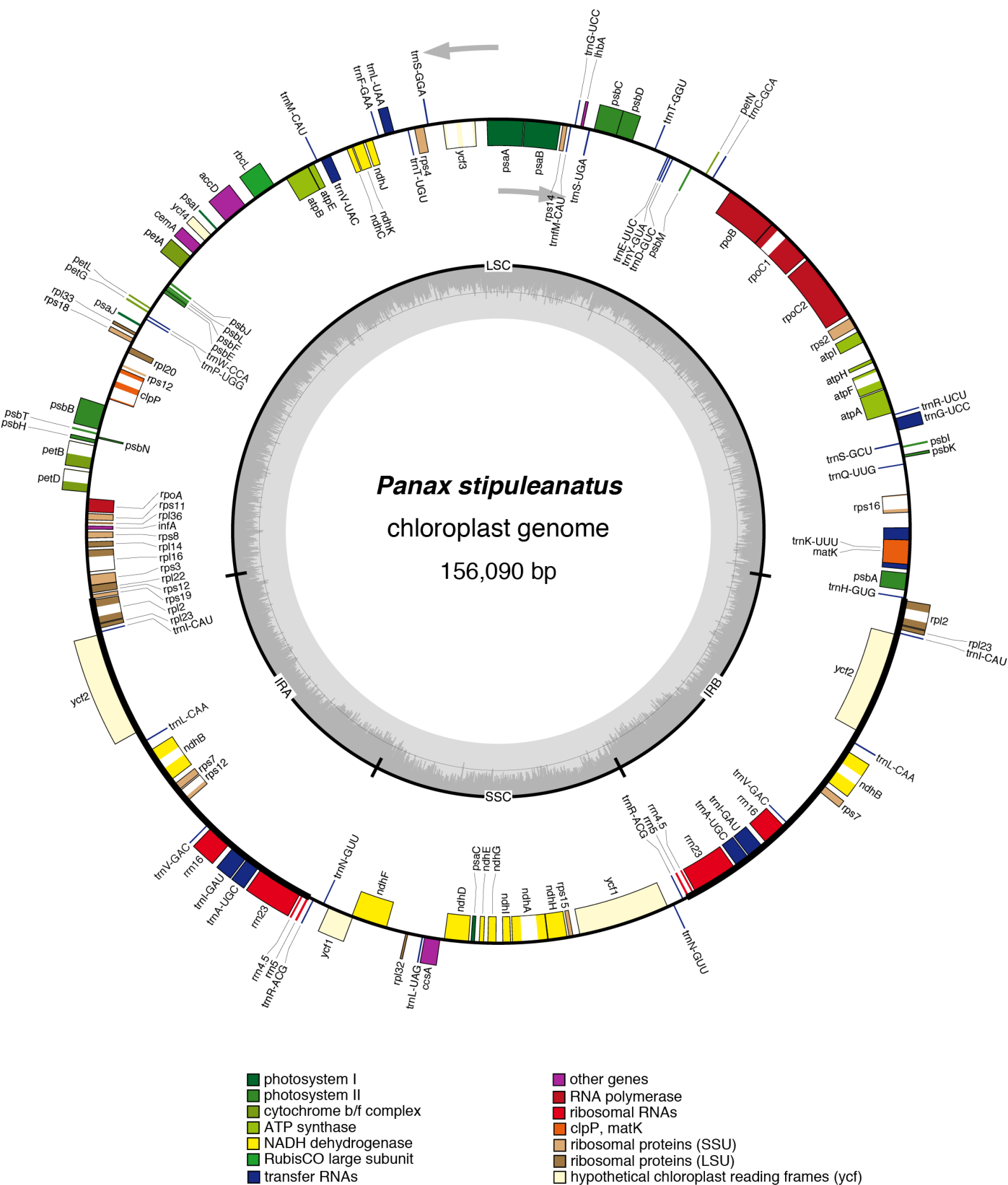

Supplement: Supplementary file 9 — Figure S5. Annotated plastid genome for P. stipuleanatus. (PDF 440 kb) [file 12862_2018_1160_MOESM9_ESM.pdf]

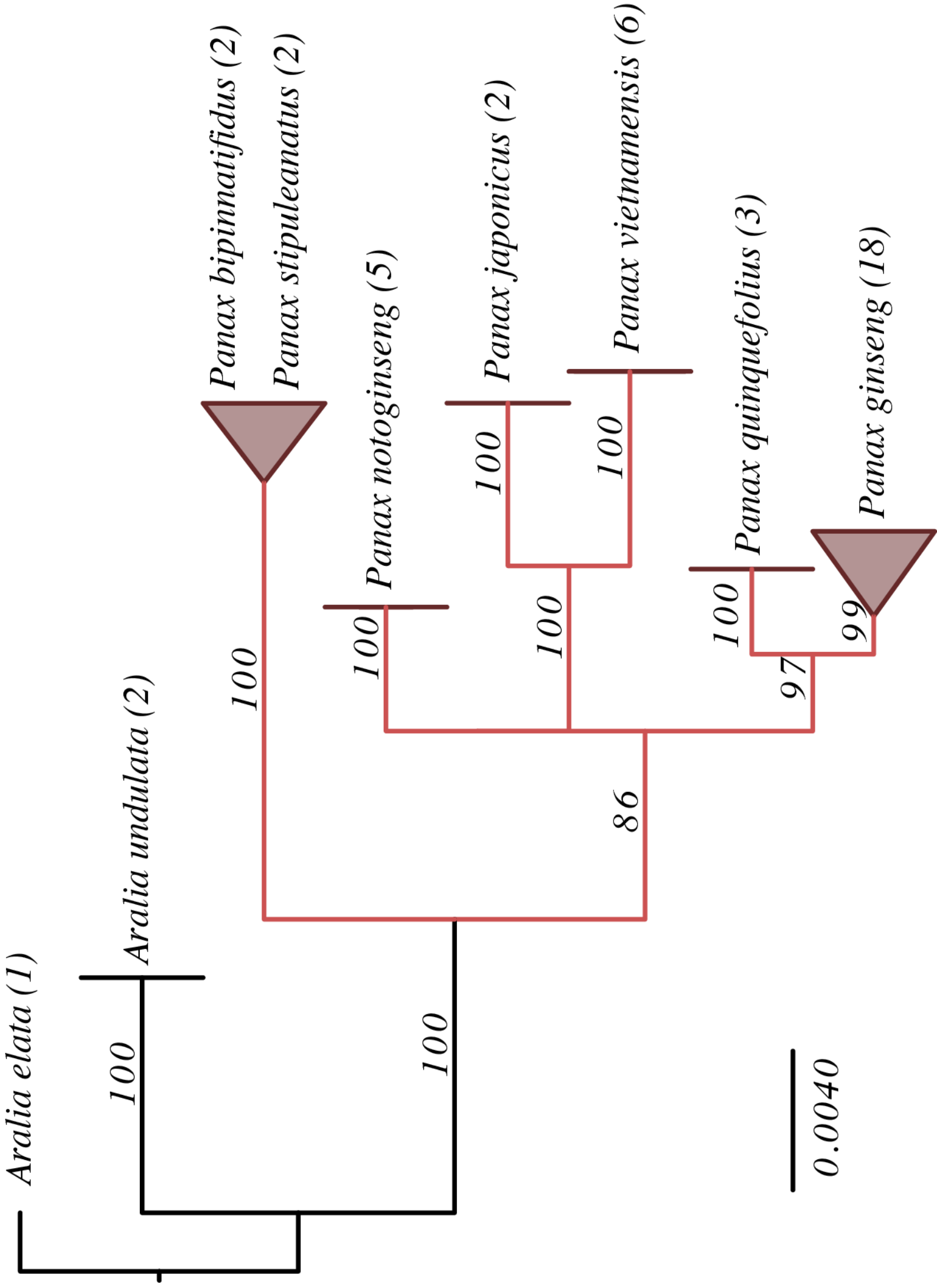

Supplement: Supplementary file 10 — Figure S7. ML phylogeny for marker trnC-rps16. The bootstrap values are represented in italic on the branches. The red branches represent supported species delimitation. (PDF 108 kb) [file 12862_2018_1160_MOESM10_ESM.pdf]

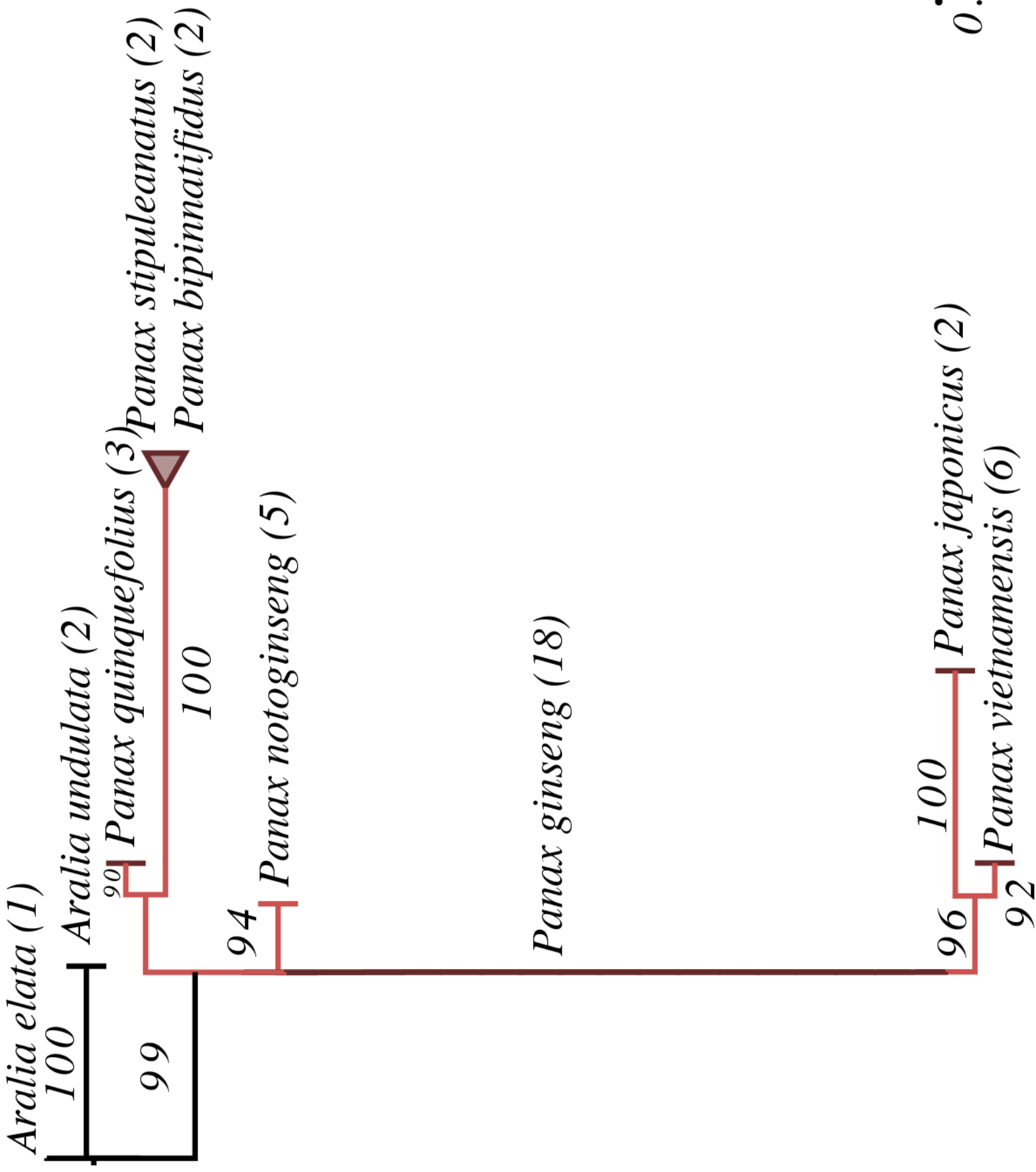

Supplement: Supplementary file 11 — Figure S8. ML phylogeny for marker trnE-trnM. The bootstrap values are represented in italic on the branches. The red branches represent supported species delimitation. (PDF 85 kb) [file 12862_2018_1160_MOESM11_ESM.pdf]

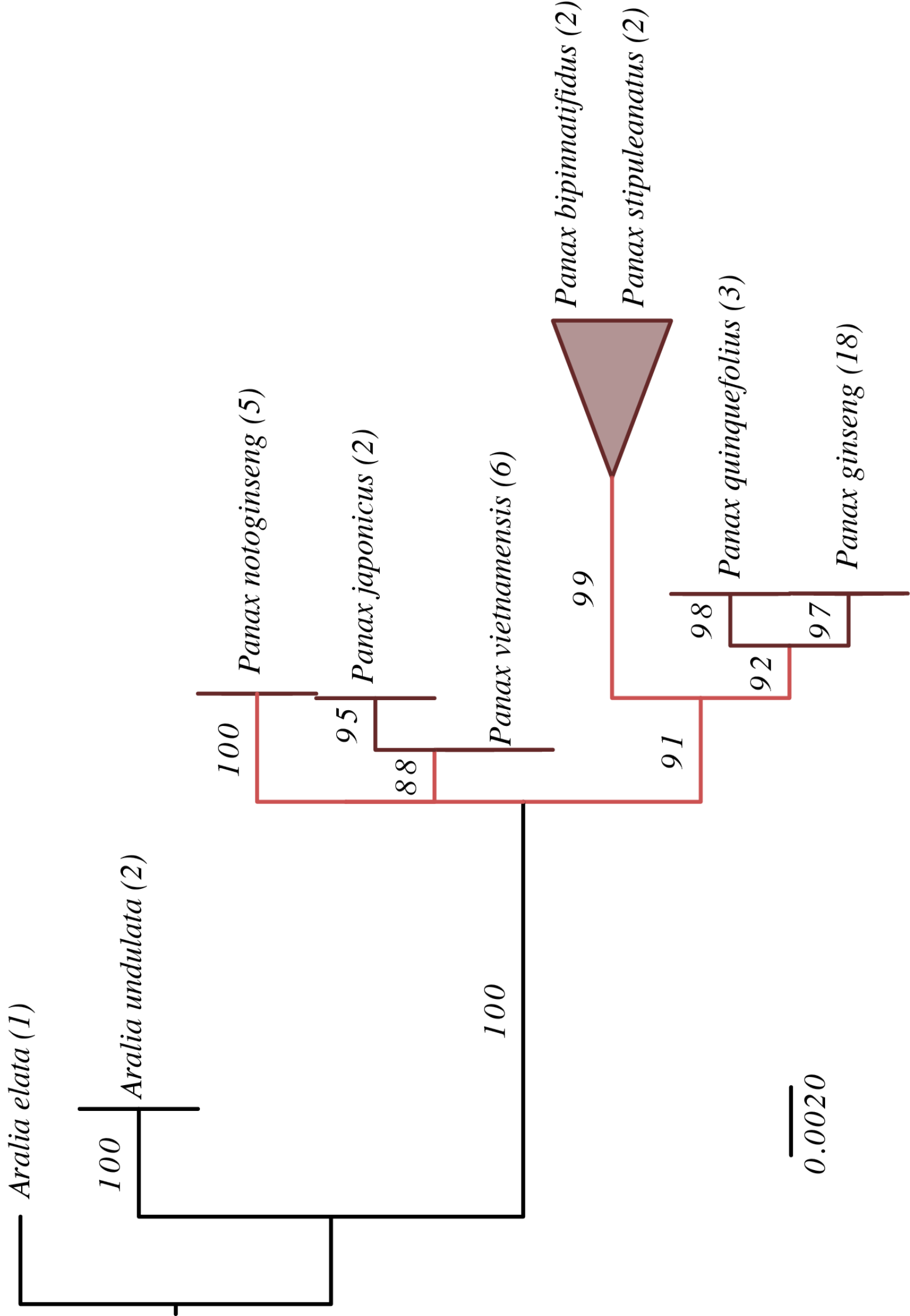

Supplement: Supplementary file 12 — Figure S9. ML phylogeny of marker trnS-trnG. The bootstrap values are represented in italic on the branches. The red branches represent supported species delimitation. (PDF 116 kb) [file 12862_2018_1160_MOESM12_ESM.pdf]

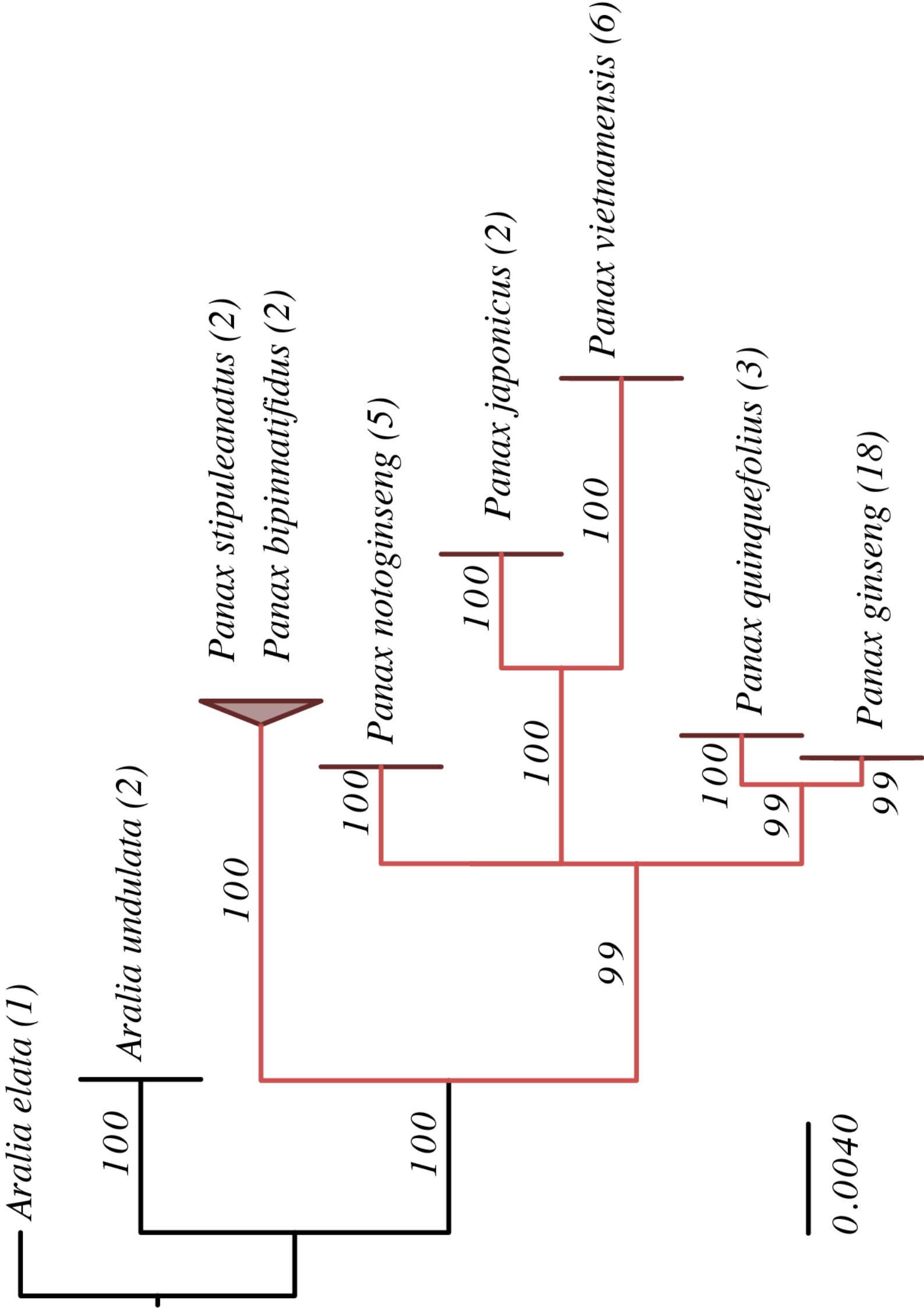

Supplement: Supplementary file 13 — Figure S10. ML phylogeny of the marker psbM-trnD. The bootstrap values are represented in italic on the branches. The red branches represent supported species delimitation. (PDF 102 kb) [file 12862_2018_1160_MOESM13_ESM.pdf]

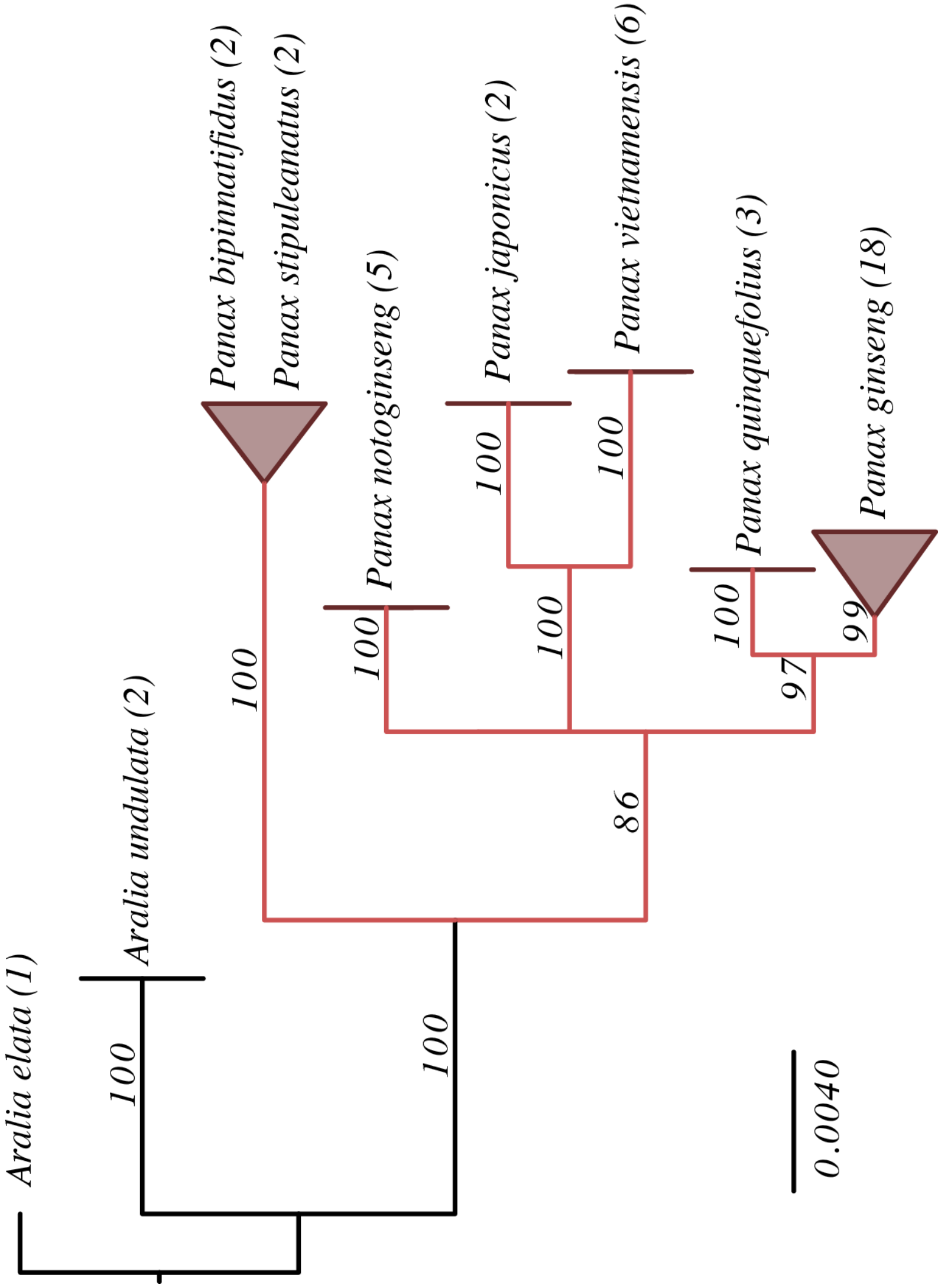

Supplement: Supplementary file 14 — Figure S11. ML phylogeny for the concatenated matrix with the four markers, trnC-rps16, trnS-trnG, trnE-trnM and psbM-trnD. The bootstrap values are represented in italic on the branches. The red branches represent supported species delimitation. (PDF 108 kb) [file 12862_2018_1160_MOESM14_ESM.pdf]

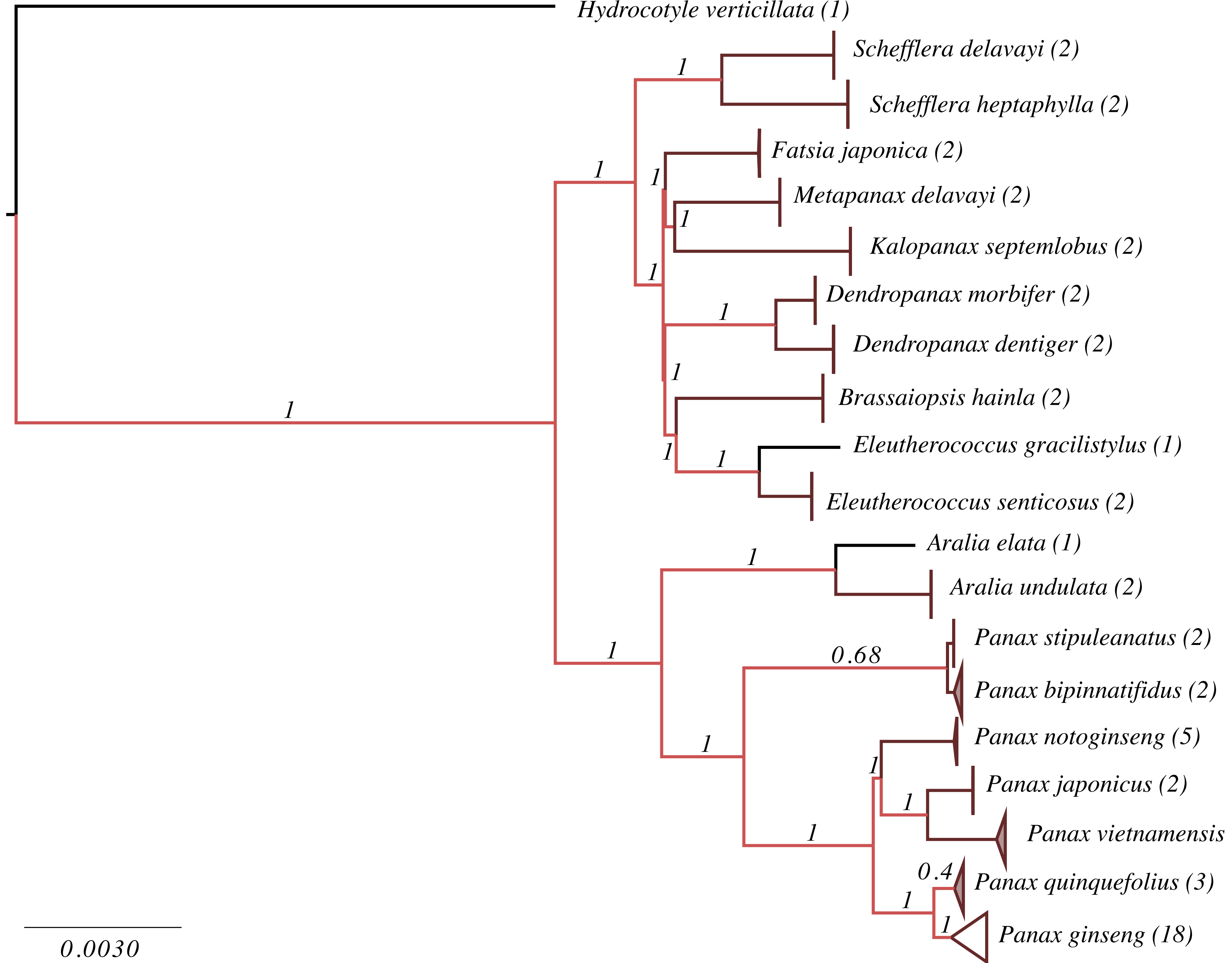

Supplement: Supplementary file 15 — Figure S6. Results of the mPTP species delimitation analysis on the full plastid genome matrix. The red lines illustrate the branches representing speciation and the brown lines the branches representing coalescence processes. The numbers on the branches represent the Bayesian posterior probabilities for the delimited species. (PDF 145 kb) [file 12862_2018_1160_MOESM15_ESM.pdf]
