# Supplementary material for: Phylogenomics and barcoding of Panax: toward the identification of ginseng species
Source: BMC Evol Biol. 2018 Apr 3;18:44. doi: 10.1186/s12862-018-1160-y (PMC5883351; doi:10.1186/s12862-018-1160-y)
Supplement: Supplementary file 8 — Figure S4. Annotated plastid genome for P. vietnamensis. (PDF 456 kb) [file 12862_2018_1160_MOESM8_ESM.pdf]

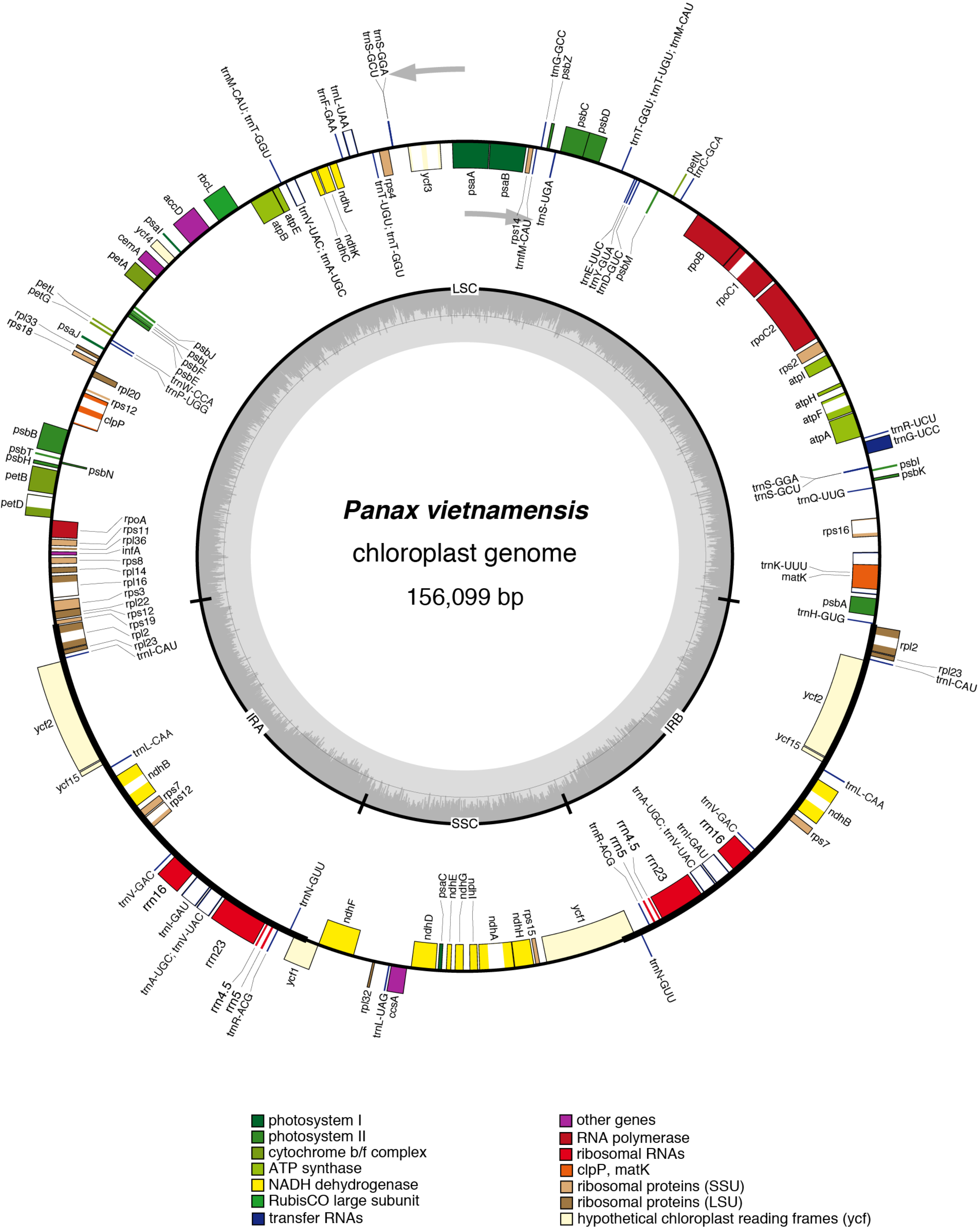

Panax vietnamensis
chloroplast genome
156,099 bp
LSC
IRA
IRB
SSC
photosystem I
photosystem II
cytochrome b/f complex
ATP synthase
NADH dehydrogenase
RubisCO large subunit
transfer RNAs
other genes
RNA polymerase
ribosomal RNAs
clpP, matK
ribosomal proteins (SSU)
ribosomal proteins (LSU)
hypothetical chloroplast reading frames (ycf)
